# Opportunities for Recent Graduates

**Published:** 1906-09-01

**Authors:** 


### Opportunities for Recent Graduates.

### POST-GRADUATE TEACHING FROM THE GENERAL PRACTITIONER'S POINT OF VIEW.

Let it be granted that never in the history of medicine has such a wealth of material for teaching or such opportunities for learning been available as at the present moment, and never were the responsible authorities more alive to the necessity for continuing the education of the qualified medical man; but it may fairly be asked, Do they fully realise his requirements?

### What are his Requirements?

The necessities of a student are sharply defined, and his teaching is easily organised, extending as it does over the five or six years; but it is a very different matter to understand and supply the wants of a qualified man in a busy practice. Although still a student, he occupies a somewhat different position by reason of his qualification. The English, and especially the London, student is characteristically conservative. He unconsciously learns to believe that it is "bad form" to seek information outside his own school; that, in fact, it would be a reflection on his teachers to do so; and further, that what his teachers do not supply is not worth troubling about. This attitude, so surprising to strangers, somewhat interferes with his early desire for extended work which the necessities of his practice soon force upon him, and so save him from the narrowness to which his conservatism may lead.

### Types of Graduates.

There are several types of qualified men who seek post-graduate work, and they admit of classification under three groups: (1) Those who wish to obtain further qualifications or degrees; (2) those who wish to devote special attention to one or more branches of their profession; (3) those who wish to amplify their experience all round, and to increase their reputation.

The needs of the first are simple and easily met. Their essentials are clearly defined by the examining Boards, and are readily met by the ample facilities afforded at the schools attached to the hospitals or affiliated to the University. The second group is not so easily dismissed, while their requirements are also sharply defined; the methods of meeting them, although ample, demand a short consideration. The phrase "devoting special attention to one branch of work" does not imply any intention of becoming a "consulting specialist"; it refers to one who in general practice, having cases in which he takes special interest, wishes to learn as much about them as he possibly can, by visiting a hospital or department specially devoted to that particular class of disease. Or, again, one who, wishing to acquire a more intimate acquaintance with the scientific aspect of his work, does so by systematically attending the different special hospitals. The one requires perhaps only a few attendances to obtain what he wants, while the other requires a systematic course adapted to his convenience and means. By attending one or two afternoons per week and taking the specialties separately, he covers a large field and acquires much sound information with small effort. Facilities based on these lines are provided not only at the special hospitals, but also by the Polyclinic. Good practical work is supplied by most of the special institutions, which are in many respects more in accord with his wants than is the case of special departments attached to the general hospitals. At nearly all of them minor appointments entailing one or two afternoon attendances per week with a certain amount of responsibility are available for three or six months. Such posts prove of great value, and, demanding but little sacrifice of time, we strongly recommend them to one who wishes to obtain a sound knowledge of the subject. He need not be frightened by visions of refraction hard labour, or tedious tuning-fork research; with the help of enthusiasm and intelligent selection he will find his time both profitably and pleasantly spent.

### The Conscientious Practitioner.

We now come to the third, the most numerous, and perhaps least understood, of the three classes—the conscientious practitioner who, alive to his own limitations, wishes to do the best for his reputation, his patients, and his pocket. His requirements are so real and so many that a detailed consideration may be pardoned. In the first place, he requires a practical experience which he can readily and directly apply to his patients' needs. He may be forgiven a not unnatural aversion to academic lectures, remembering his early student days and the work which was of the greatest service in his examination—the out-patient department. He requires that which books and the lecture theatre do not supply—namely, personal and practical acquaintance with the diseases of every-day life. In academic lectures he sees an expensive luxury which he cannot afford at present. His passive toleration of them is only equalled by his enthusiasm for a practical "tip" or a useful prescription. He enjoys a "good tip," and justifiably, feeling that he is acquiring in a concrete form the experience of a practical teacher. Laboratory and high clinical research he admires from a distance, but he welcomes a knowledge of the quickest, simplest, and most reliable method of demonstrating a "typhoid reaction," a blood count, a sugar or albumin test, or the staining of a tubercle bacillus. He sees in his own practice occasional cases of aural suppuration, but he wishes to see groups of them in different stages and degrees of severity, to become familiar with their differential diagnosis, complications, and sequelæ, and the perfected details of treatment. He wants to know when and how to syringe the ears, to inflate the tympanic cavity, how to see the glottis, and to be able to appreciate what he does see. These things he views from a business aspect, and realises that contact with a practical teacher is a "solid asset" to him which can be applied in his own practice, yielding a substantial return in cash and "kudos." He wishes to be "up to date," yet while fully appreciating the value and fascination of an expert's lecture, he prefers to absorb it through the medical journals at his leisure; but he cannot see the "cream" of a large out-patient clinic in the weekly journal. "I know," says he, "that in functional aphonia the voice may be at once restored by the interrupted current, but in my last case I did not succeed; therefore I want to learn by practical demonstration why I failed." He has no desire to become an expert laryngologist, oculist, or aurist, but he wishes to become more expert. He prefers the conversational demonstration in which he takes a direct personal share to the more impersonal lecture. Finally, he requires his teaching so arranged as to interfere as little as possible with his regular work, and at a moderate cost pecuniarily.

### The Mature Practitioner.

There is, however, another type of medical man whose requirements must be considered—namely, the one who, having matured in practice and being able to take matters more easily, realises that he is becoming somewhat "crystallised," and is in want of some clinical refreshment. He requires much the same as his younger colleague, but less of it; therefore asks for an occasional welcome in the clinical circle and an occasional lecture.

### How are their Requirements Met?

It is now fully recognised that the exigencies of private practice, the accumulation of scientific facts, and the keenness of competition render post-graduate study essential to professional success. The advertisement columns of our journals show that the demand is being promptly responded to; the wealth of opportunities presented is even such as to confuse a student in his selection, and we fear in the anxiety and enterprise of attracting students that some of the more important essentials to many of them are either overlooked or imperfectly fulfilled, particularly with regard to those forming our third group—namely, the average general practitioner. He certainly "has the run" of all the hospitals; he has the Polyclinic, with its manifold attractions; yet for him they have their limitations, and we venture to think that they do not give him exactly what he wants; therefore it is the special hospitals to which he naturally and unquestionably gravitates, for he finds them more closely adapted to his requirements, and at them he is not crowded out by junior students. Further, he finds there short courses devoted to special subjects of immediate interest to him, arranged at convenient times and at moderate fees.

### A High Standard in Illustration.

In support of our contention, we will take for illustration the method adopted at the Central London Throat and Ear Hospital, which we believe was one of the first to seriously entertain the requirements of the general practitioner in post-graduate teaching. There, courses of six practical classes are given which are limited in their admissions and held once weekly at 5 o'clock. Each one is illustrated by selected cases confined to a particular subject, such as laryngeal, aural, nasal, etc.; in fact, the cream of the surgeon's clinic. Their clinical anatomy, pathology, examination, diagnosis, and treatment are practically demonstrated in such a way that each student personally takes part in the work. Each surgeon being responsible for his own particular subject or region, overlapping and repetition are avoided. It is a system both economical and practical; therefore appeals directly to the busy student. The courses being continuous through the session admit of commencement at any stage—a great convenience to practitioners, and one which prevents overcrowding, one of the greatest obstacles to successful teaching. Included in each course are practical demonstrations of clinical pathology, which embrace the examination of discharges, tissues, sputum, etc. Further, attendance at operations and the usual routine of out-patient work is an optional yet integral part of a course in which personal co-operation is specially encouraged.

We refer to this system not in any invidious or didactic spirit, but simply for the purpose of indicating the lines upon which post-graduate teaching may prove most useful to practitioners, and one which more nearly realises their requirements. We are further of the opinion that such methods, if conscientiously carried out, can be adopted without any sacrifice of the scientific standard of teaching traditional in our schools.

Sooner or later we hope to see a grouping of hospitals, particularly the "specials"; for it is by combination and mutual co-operation that the full benefit of such a wealth of clinical material can be most economically employed.

